# Use of Rhizobacteria and Mycorrhizae Consortium in the Open Field as a Strategy for Improving Crop Nutrition, Productivity and Soil Fertility

**DOI:** 10.3389/fmicb.2019.01106

**Published:** 2019-05-21

**Authors:** Anas Raklami, Noura Bechtaoui, Abdel-ilah Tahiri, Mohamed Anli, Abdelilah Meddich, Khalid Oufdou

**Affiliations:** ^1^Laboratory of Biology and Biotechnology of Microorganisms, Faculty of Sciences Semlalia, Cadi Ayyad University, Marrakesh, Morocco; ^2^Laboratory of Biotechnology and Plant Physiology, Faculty of Sciences Semlalia, Cadi Ayyad University, Marrakesh, Morocco

**Keywords:** biofertilizers, PGPR, rhizosphere, nutrient uptake, mediterranean region

## Abstract

Plant growth promoting rhizobacteria (PGPR) and arbuscular mycorrhizal fungi (AMF) are known for their beneficial effects. In recent years, more attention has been paid to their use as biofertilizers to reduce the use of chemical fertilizers causing significant damage to the environment. To have high plant yields, biofertilizers may not be able to sustain plant demands and could be used in combination with chemical fertilizers. However, the application of biofertilizers in the field such as rhizobacteria and AMF are understudied and powerfully needed. In this context, this study aims to evaluate the effect of inoculation with rhizobacteria and AMF and their potential to stimulate two of the most economically important crops in Mediterranean semi-arid areas (*Vicia faba* L. and *Triticum durum* L.). The effect of inoculation was studied in field experiment with six treatments: (i) the control without inoculation (C), (ii) PGPR alone (PG), (iii) rhizobia alone (R), (iv) the mixture of PGPR and rhizobia (PR), (v) AMF alone (M), and (vi) the mixture of PGPR, rhizobia and AMF (PRM). The inoculation with the consortium of PGPR-rhizobia-AMF (PRM) induced the greatest effect. This inoculation improved the growth parameters (dry weight of shoots and roots) of faba bean and wheat. An improvement of 130, 200, and 78% was observed in *V. faba* shoot and root dry weight, and the number of leaves, respectively. Similarly, shoot and root dry weight and number of leaves of *T. durum* were enhanced by 293, 258, and 87%, respectively. The inoculation improved the productivity of studied plants presented by the number and weight of bean pods (270 × 10^4^ ha^-1^ and 30737.5 kg.ha^-1^) and wheat spikes (440 × 10^4^ ha^-1^ and 10560 kg.ha^-1^). In addition, the mineral analyses showed that the inoculation with PGPR-rhizobia-mycorrhizae improved N, P, Ca, K, and Na shoots contents, as well as the contents of sugar and proteins. Finally, we revealed the positive impact of the tested biofertilizers and the interest of adoption of innovative practices improving crops productivity and soil fertility.

## Introduction

The world population has recorded considerable growth accompanied by an accentuated need for food products; this puts pressure on the agricultural sector (FAO, 2011; PRB, 2017). In order to meet the high demand of agronomy food and to reduce the malnutrition risk and poverty, authorities and organizations recommend doubling agricultural production and develop food supplements (FAO, 2011). In this context, the massive use of fertilizers and plant protection products has taken place to address nutrient deficiencies and controlling diseases and weeds (Aktar et al., 2009). The intensive use of chemicals (fertilizers, pesticides) has unquestionably succeeded in increasing yields and control crops. However, in return, it has polluted the agroecosystems (water and soil) (Riah et al., 2014).

One of the ways of optimizing the use of fertilizers and plant production (maintenance of nutrients and reduction of losses) would be to valorize certain biological components of the soil, including PGPR, rhizobia, as well as the arbuscular mycorrhizal fungi (AMF). These microorganisms can directly facilitate plant growth and promote plant health by helping to acquire nutrients (nitrogen, phosphorus, and essential minerals) and/or modulating root growth and architecture through the release of plant phytohormones (auxins, cytokinins …) (Vessey, 2003). On the other hand, they indirectly diminish the inhibitory effects of various pathogens on plant development by releasing chemical compounds such as hydrogen cyanide (HCN), siderophores, antibiotics, and antifungal substances. Furthermore, they induce systemic resistance of plants or simply by spatial or temporal competition, as well as the colonization of probable sites of infection (Vacheron et al., 2013; Ahemad and Kibret, 2014). In addition, the AMF can improve water and mineral status of the plant through the transfer of water and mineral elements, especially phosphorus to the plant (Baslam et al., 2014). Indeed, the elongation of extra-radical mycelium increases the contact surface between minerals of the soil and plant roots. Therefore, they can explore inaccessible areas for the plant to collect water and nutrients and transfer them to the host plant allowing an improved growth, yield, and quality of crop production (Barea et al., 2017). The application of these microorganisms as biofertilizers in agricultural practice appears to have a significant effect on agricultural yield, particularly in *V. faba, Phaseolus vulgaris, Vigna unguiculata*, and *Trifolium alexandrinum* (van der Heijden et al., 2006; Castellanos-Morales et al., 2010; Clautilde et al., 2011; Barea et al., 2017). However, the impact of bacteria and AMF on plant growth and nutrition, in the open field, is scant, incomplete or lacking. More studies elucidating the effect of these microorganisms are strongly needed because they are difficult to monitor in the field (Hart et al., 2017; Ryan and Graham, 2018).

Accordingly, the aim of this study is to test in the field, the effects of rhizobacteria and mycorrhizae inoculation on soil fertility and on the growth, productivity, nutritional and biochemical parameters of *V. faba* and *T. durum.* These two plants are widely cultivated in Mediterranean semi-arid areas such as Morocco and especially in the Marrakesh region (MADRPM/DERD, 2011; Wahbi et al., 2016b).

## Materials and Methods

### Study Site

The study of the impact of rhizobacteria and mycorrhizae inoculation was carried out at a private farm spread over a total area of three hectares. It’s an agricultural land equipped with a drip irrigation system. The distance between drip lines for the same board (block) is 40 cm and with 15 cm as a distance between each internal dripper. The drip hose used is equipped with suitable internal drippers (sheath) which release 2 l/h. This farm is located in the Marrakesh region (Morocco) and more specifically in Tamesloht Commune (latitude of 31°54′18″ N, longitude of 8°02′08″ W, 511 m above sea level). The regional climate of the experimental site is typically Mediterranean, with 251 mm of rainfall (from September to May) and the mean air temperatures are 28.2°C in autumn, 18°C in winter, and 26°C in spring. The mean minimum and maximum annual temperatures are 7 and 32°C, respectively. The soil plots undergoing our experiment have never been cultivated, benefited or treated before by chemical fertilizers or other organic manures.

### Characterization of Rhizobacterial Strains

The rhizobacteria used for inoculation of our collection were two PGPR strains: BS17 (*Acinetobacter* sp.) and PGP27 (*Rahnella aquatilis*), and two rhizobia: RhOF4 (*Ensifer meliloti*) and RhOF155 (*E. meliloti*).

The rhizobacterial strains were tested for different PGPR activities and biochemical characteristics: tricalcium phosphate and potassium solubilization (as described by Alikhani et al. (2006), siderophores (Schwyn and Neilands, 1987), exopolysaccharides (Lee et al., 2007), indole acetic acid (Bano and Musarrat, 2003), HCN production (Lorck, 1948), and nitrogen fixation (N_2_) (Onyeze et al., 2013). As for nitrate reduction to nitrite and to dinitrogen, glucose fermentation, arginine dihydrolase, urease, gelatinase, assimilation of some sugars (glucose, maltose, mannitol), they were investigated using the API 20NE according to the manufacturer instructions (API System, Biomerieux).

### Arbuscular Mycorrhizal Fungi Inoculation

The used consortium AMF was isolated from the Tafilalet palm located at 500 km southeast of Marrakesh and it contains a mixture of native species: (i) *Glomus* sp. (15 spores/g of soil), (ii) *Sclerocystis* sp. (9 spores/g soil), and (iii) *Acaulospora* sp. (one spore/g of soil) (Meddich et al., 2015). These mycorrhizae were developed in corn roots since they cannot be grown separately from the plant. Briefly, corn seeds were disinfected and germinated inside vermiculite (previously sterilized at 200°C for 3 h) watered with sterile distilled water. After a week of germination, the corn plants were planted in plastic pots (13 cm × 09 cm) containing soil with AMF. These plants were watered regularly with distilled water with a 30 ml-weekly intake of the modified nutrient solution of Long Ashton (Plenchette et al., 1982). After 3 months of culture, the mycorrhized roots were disinfected for 10 min (Strullu, 1986), rinsed three times for 10 min with sterile distilled water and cut into fragments of 1–2 mm long.

For detection of root colonization by AMF, the roots of *V. faba* and *T. durum* were washed and cleaned up with 10% KOH at 90°C. Then, they were rinsed and suspended in lactic acid for 7–10 min at room temperature. Thereafter, the roots were stained with 0.05% trypan blue at 90°C for 20 min (Phillips and Hayman, 1970). Random fragments of 1 cm in length roots were mounted between slide and coverslip in a drop of glycerol, 15 root-fragments per slide. They were then observed under a microscope to quantify the mycorrhization frequency.

The frequency of mycorrhizae infection (percentage of root segments infection) of roots was determined by the technique described by Trouvelot et al. (1986), and was calculated as follows:

$$F\;\left(\%\right)\text{=}\frac{\left(N-N0\right)}{N}\times 100$$

with, *N* = number of observed fragments, and *N*0 = number of non-mycorrhizal fragments.

### Experimental Plan and Growth Parameters

A split plot design, with plant species as a main factor and inoculation as a sub-factor. Treatments were plant species (either faba bean or wheat grown separately) and microorganisms inoculation: (i) the control without inoculation (C), (ii) PGPR alone (BS17+PGP27) (PG), (iii) rhizobia alone (RhOF4+RhOF155) (R), (iv) PGPR and rhizobia (BS17+ PGP27+RhOF4+RhOF155) (PR), (v) AMF alone (M), and finally (vi) the mixture of PGPR + rhizobia + PRM. The dimensions of each elementary plot were 1.5 m × 0.8 m each. Each main plot was spaced 0.4 m from the next plot and spaced 1 m between the two crops (bean and wheat) to avoid any possible source of contamination. The following Table 1 illustrates the different treatments used in this field experimentation.

**Table 1:** The different treatments applied in the field experimentation.

Code	Type of inoculation	Treatment
C	Control	No inoculated plants
PG	PGPR alone	PGP27 + BS17
R	rhizobia alone	RhOF4 + RhOF155
PR	PGPR + rhizobia	PGP27 + BS17 + RhOF4 + RhOF155
M	AMF	AMF
PRM	PGPR + rhizobia + AMF	PGP27 + BS17 + RhOF4 + RhOF155 + AMF

*AMF, arbuscular-mycorrhizal fungi.*

The crops were sown in February 2017, homogenous bean seeds (Aguadulce variety) and homogenous wheat seeds (Karim variety) were disinfected with sodium hypochlorite (12°) diluted 1/3 for bean seeds and 1/5 for wheat seeds. After series of successive rinses with sterile distilled water, the seeds were germinated at 28°C for 48 h for faba bean and 24 h for wheat. After germination, sprouted seeds were inoculated with PGPR alone, rhizobia alone or a mixture of rhizobia and PGPR for 30 min in darkness for each treatment. For each bacterial strain, the optical density is equivalent to 1 at 600 nm. About 5 g of wheat (47.86 g as a weight of thousand seeds) per plot (1.5 m × 0.8 m each) were sown and 12 bean seeds (1499.16 g as a weight of thousand seeds) per plot (1.5 m × 0.8 m each) were transferred to the field in three rows separated by 0.3 m (due to four seeds per row) and inoculated with 5 ml of bacterial consortium. A second inoculation by these bacteria was scheduled 15 days after seed sowing. For treatments containing mycorrhizal fungi, bean and wheat seeds were inoculated with 2 g (fresh weight) of corn mycorrhized roots near the root system of faba bean and wheat plants. A non-inoculated control was conducted under the same conditions to determine the effect of agricultural soil native flora on the growth of both bean and wheat crops.

To evaluate the growth and the productivity performance, several parameters were measured after a 5 month-culture at the seed maturity stage: the soot and root dry weights (dried in 65°C for 3 days) and the number of leaves. The mound was taken out with all roots and their rhizosphere; for both bean and wheat, and then washed with water till having clear roots. We also measured the number of flowers at the flowering stage (April 2017).

At the seed maturity stage (the end of June 2017), the grain productivity was evaluated by the measurement of number and weight of wheat spikes and bean pods on the harvested plants randomly chosen in the middle of the plot. These measurements were recorded on a plot basis and were converted to hectare for statistical analysis.

Several studies have reported that the grain yield is followed by the determination of several parameters including the number and the weight of spikes and pods (Bozoglu et al., 2002; Olfati and Malakouti, 2013; Qiao et al., 2015; Wahbi et al., 2016b).

### Minerals and Biochemical Analyses

The mineral determination (Na, K, Ca, and phosphorus) was carried out after mineralization of plant shoots. The samples were distributed in crucibles at the rate of 0.5 g of dry matter per crucible and then placed for 6 h in the oven at 550°C. The obtained ash was added with 3 ml of 6 N HCl, evaporated on a hot plate and then recovered with hot distilled water. The obtained solutions were filtered and the extracts were collected and subsequently stored.

Phosphorus was determined according to Olsen and Sommers (1982). Na, K, and Ca elements were determined by a flame photometer (AFP 100 flame photometer). The total content of nitrogen (N) in plants was carried out according to the method described by Rodier (1984), which consists of digesting 0.5 g of plant dried matter using a digest block, then the ash was distilled with a semi-automatic distiller. Nitrogen was collected in a solution of boric acid and assayed with a solution of diluted sulfuric acid.

As for the biochemical analyses, extracts of different samples were prepared by grinding 0.5 g of the dry shoots of each sample with 10 ml of 80% ethanol. We centrifuged the extracts at 4000 rpm for 20 min. The extraction was done three times to have a final volume of 30 ml for each extract. The soluble proteins were determined according to the method Bradford (1976), total sugars were determined following the colorimetric method described by Dubois et al. (1956) and phenolic compounds (polyphenols) were determined according to the method described by Singleton et al. (1999).

### Physicochemical Soil Analyses

The soil physicochemical properties were analyzed before and after the field experimentation on samples taken near the roots after the removal of the superficial layer (0 to 15 cm). We evaluated the pH; measured on a soil suspension diluted 1/5 (v/v). Electrical conductivity was measured using a conductivity meter. Soil texture was determined by Robinson’s method (Baize, 1988). Carbon and total organic matter were measured according to the method described by Aubert (1978), which consists of the oxidation of organic matter by potassium dichromate in the presence of sulfuric acid. Total limestone was measured using Bernard’s calcimeter (Baize, 1988). The assimilable phosphorus was measured by the method of Olsen and Sommers (1982). Lastly, nitrogen was determined according to the method described by Rodier (1984).

### Statistical Analyses

Results are means ± SD of fifteen determinations in growth and productivity parameters (shoot and root dry weight, number of leaves and flowers, number and weight of bean pods, and wheat spikes), and four determinations in mineral and biochemical analyses. Differences among treatments were assessed by one-way ANOVA; the averages were compared by the SNK test (Student, Newman, Keuls). Significant differences at *p* < 0.05 are indicated by different letters. Growth, nutrition and yield parameters and their correlation with treatments were subjected to principal component analyses (PCA) using XLStat software.

## Results

### Characterization of Rhizobacterial Strains

The rhizobacterial tested strains showed significant PGPR activities (Table 2). They can solubilize tricalcium phosphate and potassium, with significant phosphate solubilization recorded in the case of RhOF4 and RhOF155, and important potassium solubilization was noted for BS17 followed by RhOF4.

**Table 2:** Characteristics of the tested rhizobacteria.

Activity	PGP27	BS17	RhOF4	RhOF155
Phosphate solubilization	+	++	+++	+++
Potassium solubilization	+++	+	++	+
Exopolysaccharide production	22.65	10.67	72.35	176.02
(mg of CR/OD_600_)
Siderophore production	-	-	-	-
AIA production (μg/ml)	38.07	10.76	112.43	290.64
HCN production	-	-	-	-
Nitrogen fixation	++	+	+++	+++
Reduction of nitrate to nitrite	+	+	-	-
Reduction of nitrate to dinitrogen	+	+	-	-
Glucose fermentation	+	+	-	-
Arginine dihydrolase	-	+	-	+
Urease	-	+	-	+
Gelatinase	+	-	-	-
Assimilation of glucose	+	+	+	-
Assimilation of mannitol	-	+	+	+
Assimilation of maltose	+	+	-	+

*+, Low; ++, medium; +++, High (for phosphorus and potassium solubilization and nitrogen fixation); -, absent; +, presence (for other activities); CR, Congo Red.*

Furthermore, rhizobacterial strains tested were able to produce exopolysaccharides up to 176.02 mg of CR/OD_600_ (RhOF155) and AIA from 10.76 to 290.64 μg/ml observed for BS17 and RhOF155, respectively. No strain was able to produce siderophores and HCN. RhOF4 and RhOF155 were able to fix N_2_ better than BS17 and PGP27. In addition, BS17 and PGP27 had the ability to reduce nitrate to nitrite and to dinitrogen. BS17 and PGP27 were the strains able to ferment glucose. Furthermore, the gelatinase activity seemed to be present only in PGP27 strain.

### Assessment of AMF Colonization

The results of the mycorrhizal parameters revealed that bean and wheat plants inoculated with AMF (M and PRM treatments) had a mycorrhizal frequency greater than 90% compared to non-mycorrhizal plants (Tables 3, 4). It showed the efficiency of the selected mycorrhizal consortium to establish a symbiotic relationship with the studied host plants. The plants not inoculated with AMF exhibited a mycorrhizal frequency of 35% up to 60% due to the presence of native AMF in the agricultural soil.

**Table 3:** Effect of bacteria and AMF inoculation on growth and biochemical parameters of faba bean.

	Treatments					
	C	PG	R	PR	M	PRM
Mycorrhization frequency (%)	35 (10) c	55 (9) c	56.66 (9) b	60 (9) b	95 (8) a	97 (8) a
Shoot dry weight (g/plant)	56.47 (3.42) e	74.62 (2.64) e	80.35 (4.24) c	82.32 (10.25) d	111.47 (12.86) b	129.95 (10.10) a
Root dry weight (g/plant)	7.32 (0.22) d	8.23 (0.23) c	12.21 (0.45) c	10.80 (0.55) c	15.77 (0.77) b	22.21 (0.33) a
Leaves number/plant	82.8 (1.09) e	101.2 (5.60) d	130.2 (6.61) c	108.0 (0.81) d	142.2 (4.60) b	155.0 (2.34) a
Flowers number/plant	43.0 (4.58) f	53.8 (3.83) e	61.4 (5.63) d	71.2 (4.43) c	86.6 (1.14) b	109.0 (4.74) a
Sugar content (mg eq glucose. g^-1^ DW)	1051,8 (53.15) d	1152,7 (56.07) d	1158,8 (94.34) d	1355,7 (45.56) c	1532,6 (58.61) b	1822,6 (90.25) a
Protein content (mg eq albumine bovine.g^-1^ DW)	128,46 (4.07) d	146,25 (14.37) d	279,41 (17.14) c	284,26 (18.03) c	328,46 (23.82) b	485,88 (18.60) a
Polyphenol content (mg eq gallic acid /g DW)	283,87 (12.15) a	241,00 (18.92) b	205,54 (1.78) c	192,73 (1.38) c	243,09 (3.77) b	237,19 (16.27) b

*Means ( ± standard deviation) within the same column followed by different letters are significantly different according to the Student, Newman–Keuls test at *p* < 0.05. C, control; PG, PGPR alone; R, rhizobia alone; PR, PGPR-rhizobia; M, AMF alone; PRM, PGPR-rhizobia-AMF.*

**Table 4:** Effect of bacteria and AMF inoculation on growth and biochemical parameters of wheat.

	Treatments					
	C	PG	R	PR	M	PRM
Mycorrhization frequency (%)	35 (9) c	50 (12) bc	60 (10) b	45 (7) bc	93.33 (6) a	93.33 (6) a
Shoot dry weight (g/plant)	2.56 (0.23) d	3.57 (0.50) c	6.32 (0.50) b	6.43 (0.26) b	5.81 (0.79) b	10.08 (0.59) a
Root dry weight (g/plant)	2.47 (0.75) c	3.03 (0.39) c	6.1 (0.37) b	6.9 (0.71) b	5.59 (1.04) b	8.85 (0.47) a
Leaves number/plant	26.0 (1.41) f	34.5 (1.29) e	37.75 (0.50) d	44.0 (0.81) b	40.5 (1.29) c	46.5 (1.91) a
Sugar content (mg eq glucose.g^-1^ DW)	773.98 (14.5) e	904.07 (47.2) d	1161.21 (32.8) c	1121.70 (36.5) c	1297.38 (126.8) b	1462.12 (47.4) a
Protein content (mg eq albumine bovine.g^-1^ DW)	2.97 (0.00) d	3.15 (0.09) c	3.28 (0.03) b	3.23 (0.04) bc	3.21 (0.00) bc	3.34 (0.04) a
Polyphenol content (mg eq gallic acid /g DW)	4.06 (0.08) bc	3.24 (0.06) d	4.79 (0.42) a	3.92 (0.17) c	4.41 (0.19) b	2.79 (0.11) e

*Means ( ± standard deviation) within the same column followed by different letters are significantly different according to the Student, Newman–Keuls test at *p* < 0.05. C, control; PG, PGPR alone; R, rhizobia alone; PR, PGPR-rhizobia; M, AMF alone; PRM, PGPR-rhizobia-AMF.*

### Measurement of Growth Parameters and Productivity

The shoots and roots dry weight were improved for all treatments (single, double, and triple inoculation), with a significant difference at 0.05 between the different treatments (Tables 3, 4). The greatest effect was recorded for the mixture PRM which significantly improved plant dry weight. This inoculation increased the biomass two times for faba bean and four times for wheat in comparison to the uninoculated plants. In addition, the biomass of crops was also enhanced by the other treatments regarding the control. Furthermore, the number of leaves was generally improved in the different plant inoculated compared to the control (Tables 3, 4). An average of 155 and 46.5 leaves/plant were produced in bean and wheat plants, respectively, in the PRM treatment (PGPR-rhizobia-mycorrhizae).

It was the treatment that offered more foliar production followed by an important production recorded, respectively, in M and R treatments for bean, PR and M treatments in case of wheat plants (Tables 3, 4). In terms of flower productivity, the different treatments tested enhanced the number of flowers produced by bean plants, with a higher improvement using the triple inoculation (PRM) with an average of 109 flowers/plant (Table 3).

The productivity evaluated by the fruits number as well as the weight of bean pods and wheat spikes were also improved by the differently tested biofertilizers (Table 5). Plants inoculated with the combination PRM showed the best productivity with 270 × 10^4^ pods per ha and 440 × 10^4^ spikes per ha. The weights of faba bean pods and wheat spikes were 30737.5 and 10560 kg.ha^-1^, respectively.

**Table 5:** Effect of bacteria and AMF inoculation on pod number and green pod weight of faba bean, and on spike number and spike dry weight of wheat.

	*V. faba*			*T. durum*		
	Pod number	Pod weight	Increase pod weight	Spike number	Spike weight	Increase spike weight
Treatment	per ha ( × 10^4^)	(kg ⋅ ha^-1^)	over control (%)	per ha ( × 10^4^)	(kg ⋅ ha^-1^)	over control (%)
C	105.0 (6.4) e	6857.5 (370.1) d	–	180 (40) e	4344 (741.0) c	–
PG	134.0 (26.0) d	8357.5 (203.0) d	21	260 (40) bc	5232 (486.3) c	20
R	186.0 (21.9) c	15345.0 (2127.8) b	123	260 (40.2) bc	7080 (1067.2) b	63
PR	142.5 (9.5) d	12837.5 (1624.8) c	87	340 (40) b	7008 (514.0) b	61
M	222.5 (15) b	17625.0 (1587.1) b	157	300 (76.5) b	5504 (1691.1) bc	26
PRM	270.0 (8.1) a	30737.5 (1526.2) a	348	440 (46.1) a	10560 (678.8) a	143

*Means ( ± standard deviation) within the same column followed by different letters are significantly different according to the Student, Newman–Keuls test at *p* < 0.05. C, control; PG, PGPR alone; R, rhizobia alone; PR, PGPR-rhizobia; M, AMF alone; PRM, PGPR-rhizobia-AMF.*

The inoculation R and PR led to a yield increase of 123 and 87%, respectively, for faba bean, and 63 and 61%, respectively, for wheat. While the rhizobial inoculation (R) and the AMF inoculation (M) were effective on faba bean yield with 123 and 157% of pod weigh increase over the control (Table 5).

### Minerals and Biochemical Analyses

**FIGURE 1:**
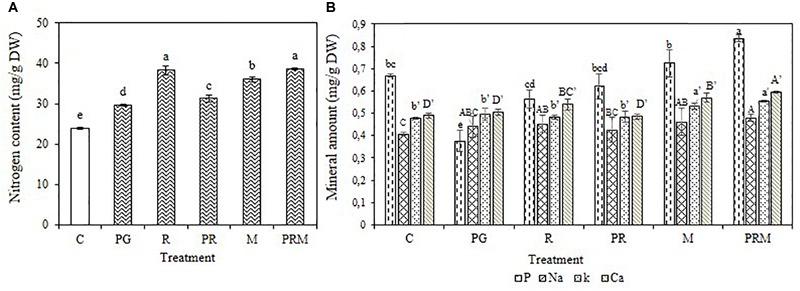
Nitrogen content **(A)** and Mineral amount **(B)** in mg/g of dry matter of *V. faba* submitted to different treatments: C, control; PG, PGPR alone; R, rhizobia alone; PR, PGPR-rhizobia; M, AMF alone; PRM, PGPR-rhizobia-AMF. Means ( ± standard deviation) within the same graphic followed by different letters are significantly different at *p* < 0.05.

**FIGURE 2:**
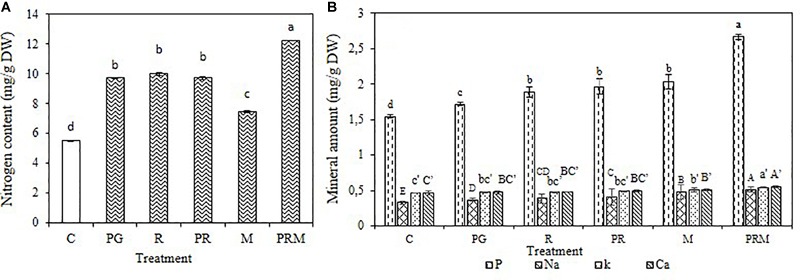
Nitrogen content **(A)** and Mineral amount **(B)** in mg/g of dry matter of *T. durum* submitted to different treatments: C, control; PG, PGPR alone; R, rhizobia alone; PR, PGPR-rhizobia; M, AMF alone; PRM, PGPR-rhizobia-AMF. Means ( ± standard deviation) within the same graphic followed by different letters are significantly different at *p* < 0.05.

The mineral status of the plants was improved independently of the inoculation by bacteria or mycorrhizae, single, double, or triple inoculation (Figure 1, 2). The consortium PGPR-rhizobia-mycorrhizae was recorded as the most efficient inoculation, with nitrogen shoots content that exceeds 30 mg/g DW (Dry Weight) for *V. faba* and 10 mg/g DW for *T. durum*. Furthermore, shoot phosphorus content was also enhanced in inoculated plants. The phosphorus content was greater than 0.8 mg/g DW for bean plants and was greater than 2 mg/g DW for wheat plants, was recorded in inoculated plants with PGPR-rhizobia-mycorrhizae as being the best treatment. In addition to nitrogen and phosphorus, this treatment was able to improve sodium concentration up to 0.48 mg/g DW compared to the control (0.40 mg/g DW) in beans and up to 0.51 mg/g DW versus 0.32 mg/g DW for the control wheat plants.

The mineral status of plants in potassium was also enhanced by treatments with better enhancement recorded in PRM and M treatments with 0.55 and 0.53 mg/g DW, respectively, for bean plants and 0.54 and 0.50 mg/g DW, respectively, for wheat plants. Calcium was also improved in plants inoculated with PRM strains followed by inoculation with mycorrhizae alone.

The inoculation of plants proved their ability to improve the sugar content in plants (Tables 3, 4). The best improvement was noted in the plants inoculated by PRM followed by plants treated with AMF (M) and double inoculation PGPR-rhizobia (PR) in both types of plants. Similarly, all treatments have improved the protein concentrations in plant shoot, except for bean inoculated by PGPR alone (Tables 3, 4).

The proteins content reached a maximum value of 485.67 mg of bovine albumin/g DW in bean and of 3.43 mg of bovine albumin/g DW in wheat. The triple inoculation PGPR-rhizobia-mycorrhizae was the best treatment that improved the proteins concentrations in the two plants. It should be noted that inoculation PRM combination improved the proteins content by up to four times more than the non-inoculated control in beans and a significant increase in the case of wheat.

As for the biosynthesis pathways of polyphenols (Tables 3, 4), they generally decreased in the inoculated plants compared to the uninoculated control. In addition, the maximum content of polyphenols was recorded in non-inoculated plants in the case of beans (283.86 mg eq gallic acid/g DW). In wheat, the lowest content of polyphenols was recorded in PG and PRM treatments.

### Principal Component Analyses

The PCA showed that inoculation treatments (in blue) and variables (in red) were correlated with degrees of variability F1: 82.11% for *V. faba* and 81.68% for *T. durum*.

**FIGURE 3:**
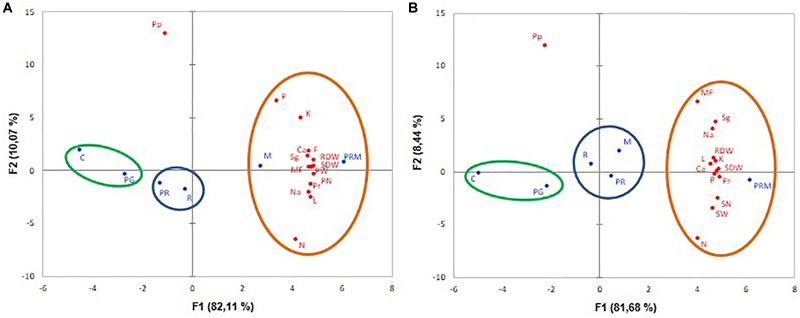
Principal component analyses (PCA) of *V. faba* **(A)** and *T. durum* **(B)** submitted to different treatments: C, control; PG, PGPR alone; R, rhizobia alone; PR, PGPR-rhizobia; M, AMF alone; PRM, PGPR-rhizobia-AMF. The growth yield and nutrition variables are represented in red. The six treatments are given in blue. MF, mycorhization frequency; RDW, root dry weight; SDW, shoot dry weight; L, leaves number; F, flowers number; N, nitrogen content; P, phosphorus content; Na, sodium content; K, potassium content; Ca, calcium content: Sg, sugar content; Pr, protein content; Pp, polyphenol content; PN, pod number, PW, pod weight; SK, spike number, and SN, spite weight.

The PCA concerning *V. faba* showed that treatments with higher growth, nutrition and yields were on the right, they corresponded to tripartite inoculation (PRM) and inoculation with mycorrhizae alone (M) (Figure 3A). Lower growth, nutrition, and yield levels were on the left, they corresponded to the control without inoculation followed by PG treatment (inoculation with PGPR bacteria). On the vertical axis, inoculation with rhizobia (R) and inoculation with PGPR+rhizobia (PR) corresponded to intermediate yield levels (Figure 3A). As for wheat, PRM treatment noted on the right, displayed the best development, as well as nutrition and productivity (Figure 3B). M, PR, and R treatments positioned in the vertical axis showed intermediate enhancement. The non-inoculated plants (C) situated on the left showed the lower correlation with the growth, nutrition and yield variables (Figure 3B).

### Physicochemical Soil Analyses

Soil physicochemical analyses carried out before the experimentation are shown in Table 6. The percentage of sand and silt was important compared to the other soil elements, which, respectively, represented 67.04 and 16.34%. According to the triangle of textures, the soil was sandy-silty. It is classified as calcareous soil with a pH higher than 7. The conductivity was 138.3 μS/cm and the percentage of limestone found was 5%. In addition, the content of total carbon (0.5%) and organic matter (0.86%) reflected that the soil was poor in organic matter. Furthermore, the soil before the applied treatments has an amount of 9.98 mg/g of nitrogen and 57 ppm of assimilable phosphorus.

**Table 6:** Soil physicochemical characteristics before the experiment.

				Total	Total	Organic	Total	Assimilable
			Conductivity	limestone	carbon	matter	nitrogen	phosphorus
Analyses	Texture	pH	(μS/cm)	(%)	(%)	(%)	(mg/g)	(ppm)
Properties of the soil	Sandy-silty	8.12	138.3	5.04	0.5	0.86	9.98	57

Soil analyses for each treatment after experimentation (Table 7) showed that the performed treatments have improved soil quality compared to the initial state. Our treatments improved the amount of the total organic matter as well as the total carbon. The greatest improvement was noted in the PRM treatment with an organic matter percentage of 3.97 and 2.30% for the total carbon followed, respectively, by R and PG treatments.

**Table 7:** Soil physicochemical characteristics after the experiment.

			Total	Organic	Total	Assimilable
		Conductivity	carbon	matter	nitrogen	phosphorus
Analyses	pH	(μS/cm)	(%)	(%)	(mg/g)	(ppm)
C	8.05	506.66	1.22	2.10	18.2	57
PG	7.18	191.30	2.20	3.79	22.4	83
R	7.18	243.33	2.27	3.91	33.6	90
PR	7.17	156.30	2.22	3.83	29.4	60
M	7.67	435.66	2.17	3.75	28.0	49
PRM	7.38	204.50	2.30	3.97	40.6	34

*C, control; PG, PGPR alone; R, rhizobia alone; PR, PGPR and rhizobia; M, AMF alone; PRM, PGPR, rhizobia and AMF.*

The total nitrogen content in the soil was also enhanced by the applied treatments. A significant improvement was observed in the triple inoculated (PRM) plant rhizosphere with an average of 40.6 mg of N/g. With regard to the assimilable phosphorus, we noted a better improvement in the soil of the R and PG treatments. In addition, a lower amount was noted in the M and PRM treatments having, respectively, 49 and 34 ppm.

## Discussion

Growth promoting rhizobacteria and AMF are the most important plants’ symbionts. They can play a crucial role in natural ecosystems and stimulate plant growth, productivity, and nutrition through several mechanisms. They may not only improve nutrient acquisition (Bhardwaj et al., 2014) but they also increase the strengthening of root architecture (Gamalero et al., 2004) and the inhibition of pathogens (Baslam et al., 2014; Goswami et al., 2016). In sustainable agricultural cropping systems, these characteristics can be of crucial importance since they are based on biological processes to maintain soil fertility, plant development, and productivity.

Characterization tests of our rhizobacterial strains showed that they have PGPR activities and they may improve plant growth and development by providing essential nutrients such as nitrogen, phosphorus, and potassium. The weak concentrations of nutrients limit the growth of and the productivity of the crops (Shen et al., 2016). Moreover, the studied rhizobacteria produce auxin that can modulate growth and root architecture (Gamalero et al., 2004), and exopolysaccharides that may solubilize phosphate and maintain water film necessary for photosynthetic activity and plant growth (Lee et al., 2005; Sharma et al., 2013; Tarraf et al., 2017). The findings of API 20NE showed that our tested strains were able to metabolize several compounds that can be produced by plant roots (amino acids, carbohydrates, and organic acids).

Furthermore, our results showed a mycorrhization frequency higher than 90%, this translates our mycorrhizal consortium capacity to infect the tested plants’ roots. The infection frequency is considered among the critical parameters of plant-mycorrhizal symbiosis since it expresses the importance of the root infection.

Hence, the studied microorganisms were good candidates to test on plant yield. The synergy between bacteria and mycorrhizal fungi led to a stimulation of spore germination, mycorrhizal root colonization, and increase in total bacterial populations (Artursson et al., 2006). This synergy causes an increase in nitrogenase activity in the case of nitrogen-fixing bacteria, which contribute to better fixation of atmospheric nitrogen (Barea et al., 2002; Jia et al., 2004). It has been found that nitrogen-fixing capacity is enhanced in mycorrhizal plants (Karandashov and Bucher, 2005).

We noted that the field inoculation with the tested microorganisms improved growth, nutrition, and productivity of bean and wheat plants, compared to the uninoculated control. All the treatments were beneficent for *V. faba* and *T. durum* plants. The best treatment was the inoculation with PGPR-rhizobia-mycorrhizae. A comparable growth enhancement effect of bean and wheat development was reported, respectively, by Jia et al. (2004) and Abbasi et al. (2011) inoculated with rhizobia, AMF and PGPR. The inoculation by rhizobacteria was beneficent for *V. faba* cultivated in pots outdoor environment (Jia and Gray, 2008), for *V. fab*a in a greenhouse experiment (Hamaoui et al., 2001), and for *P. vulgaris* in a greenhouse experiment (Zafar et al., 2012). Accordingly, Abd-Alla et al. (2014) have highlighted that rhizobia-mycorrhizae inoculation showed better improvement of *V. faba* dry weight compared to simple inoculation with mycorrhizae. However, Wahbi et al. (2016a) found that mycorrhizal inoculation had a significantly positive effect on the shoot dry weights and total shoot N in faba bean, but not in wheat.

In terms of productivity, growth improvements may lead to a better yield. The obtained results showed that the inoculation with appropriate microorganisms enhanced the growth of faba bean and wheat. This improvement of growth and nutrition is accompanied by better productivity qualitatively and quantitatively. The great yield was noticed in the case of plants inoculated with both bacteria and AMF. Our results are in accordance with those obtained by Osman et al. (2010) who showed that the inoculation of bean plants with rhizobia and PGPR improved the yield of this crop cultivated in the field. Similarly, greenhouse experimentation conducted on wheat (Abbasi et al., 2011) and field experiment conducted in Dongola university farm (Sudan) on faba bean (Osman et al., 2010), have shown the fitness of bacteria and/or mycorrhizae to improve the plant production quantitatively and qualitatively. In the same way, Qiao et al. (2015) have reported that spike and pod weights were improved by AMF inoculation. Inoculation greatly enhanced pod weights of faba bean by an increase of 407.9% in comparison to the uninoculated plants at harvest. Youseif et al. (2017) have also shown that inoculation with *Rhizobium leguminosarum* and *Agrobacterium tumefaciens* increased significantly the yield of faba bean evaluated by the number of pods per plant. Furthermore, Diaz-Zorita and Fernandez-Canigia (2009) proved that inoculation of wheat with *Azospirillum brasilense*; improved plants yield with a production of 474 spikes per m^2^ versus 434 spikes per m^2^ in the control plants.

In our study, the inoculation with bacteria and/or mycorrhizae improved the mineral content (N, P, K, Na, and Ca) in plant shoots. It is important to note that the tested rhizobacteria were able *in vitro* to solubilize phosphate, potassium, and to produce auxin and exopolysaccharides that might improve plant nutrition. Besides the PGP activities, the improvement in the mineral amount is also linked with the ability of mycorrhizal hyphae to explore more area that is not explored by plants roots and the capacity of AMF to transfer nutrients to plant roots. Indeed, the highest amount of mineral nutrient was interrelated with higher mycorrhization frequency in case of plants inoculated with our mycorrhizae inoculum. In harmony with our results, Abbasi et al. (2011) have also reported that the inoculation of wheat with PGPR led to an improvement in phosphorus and nitrogen contents three times more than uninoculated control, as well as a 58% promotion for potassium. Whereas Wahbi et al. (2016a) showed that inoculation with AMF improved nitrogen content in both faba bean and wheat cultivated in pots. It is well known that AMF are able to solubilize phosphate and to mobilize other nutrients for the plant benefit (Baslam et al., 2014; Tarraf et al., 2017). In addition, extra-radical mycelium elongation increases the contact area between soil minerals and roots. As a result, they can explore areas that are not accessible to the roots in order to collect water and nutrients and transfer them to the host plant (van der Heijden et al., 2006; Castellanos-Morales et al., 2010).

Research by Jia et al. (2004) also highlighted the influence of AMF and rhizobia on nitrogen and phosphorus accumulation in *V. faba*. In addition, it has been estimated that about 80% of the phosphorus absorbed by a mycorrhizal plant is supplied by the fungus (Marschner and Dell, 1994). Lodwig et al. (2003) have also reported that the bacteria-fungi association improved the efficiency of plants to absorb phosphorus and other nutrients.

The biochemical analyses showed that the inoculation of *V. faba* and *T. durum* with the tested microorganisms enhanced not only plant growth and nutrient uptake but also the biochemical parameters (sugar and proteins). These results are consistent with the study conducted by Al-garni (2006) who reported the increase of sugar levels in mycorrhizal soybean and *Phragmites australis* under salt stress condition. Furthermore, Clautilde et al. (2011) reported that the inoculation with *Rhizobium* and mycorrhizae increased the protein content in cowpea leaves. Shaukat et al. (2006) also revealed that inoculation with bacteria alone could improve protein content in wheat plants compared to non-inoculated plants.

Polyphenols are chemical weapons synthesized against oxidative stress to rid the cell from reactive oxygen species (ROS) generated by different stresses. Microorganisms could alleviate different abiotic and biotic stresses (Ahemad and Kibret, 2014). Our result is in agreement with that found by Baslam et al. (2014). They proved that the content of soluble phenolic compounds was lower in the inoculated treatment in comparison to the control under drought stress. Additionally, the soil physicochemical characterization was carried out before and after the experiment in order to evaluate the inoculation effect carried out on soil fertility. Soil physicochemical properties changed after culture’s harvest. The obtained results showed that all treatments had an important effect on the nutritional and physicochemical soil properties. Indeed, a high amount of nitrogen and assimilable phosphorus in rhizosphere soil of plants inoculated with rhizobacteria can be related to nitrogen fixation as well as higher phosphate solubilization activity of the tested bacteria. On the other hand, assimilable phosphorus was lower for the mycorrhizae treatments (M and PRM) than at the beginning of the experiment. This result can be explained by the fact that AMF efficiently and directly take up to the plant the macro and micronutrients such as N, K, Mg, Cu, Zn, Fe, Mn especially when present in fewer amounts in soils (Mantelin and Touraine, 2004; Meding and Zasoski, 2008).

The improvement of organic matter and carbon in soils might be correlated with the ability of our strains to metabolize different compounds produced by plants roots (amino acids, carbohydrates, and organic acids). Caravaca et al. (2002) showed that mycorrhizal inoculation of *Olea europaea* young seedlings was very effective in improving soil quality. Other studies confirmed the development and the ability of microorganisms especially PGPR and rhizobia, to improve the organic and nutrient quality of the rhizosphere soil through different mechanisms such as symbiotic and free nitrogen fixation, the production of siderophores and exopolysaccharides, the solubilization of phosphate and potassium, as well as other additional mechanisms (Lee et al., 2005; Bhattacharyya and Jha, 2012; Sharma et al., 2013).

## Conclusion

This research purpose was to test the rhizobacterial and mycorrhizal inoculation effect on growth, production, mineral nutrition and biochemical parameters of *V. faba* and *T. durum*. Based on the results of this field study, the rhizobacterial strains were able to solubilize phosphate, potassium, produced auxin and exopolysaccharides and they had the ability to colonize the rhizosphere. The selected mycorrhizal consortium showed a greater ability to infect bean and wheat plant roots. The tested symbiotic combinations improved growth, leaf production, plant shoots, and roots compared to uninoculated plants. In addition, crop yield was also increased through the high number and weight of bean pods and wheat spikes. Furthermore, the content of sugars and proteins were also improved by the biological inoculation for both plants. The obtained results have also shown that the treatments carried out have reduced the polyphenols content in plants. The overall results of this experiment demonstrated that rhizobacteria and mycorrhizal inoculums seemed to be adapted with the soil native microflora in the open field. Moreover, the biological treatments appeared to have an important effect on the soil physicochemical properties. The best treatment selected from this field experimentation was the tripartite combination comprising the mixture PRM. Field-scale experiments should be repeated over the years and in a different environment to validate the findings.

## Author Contributions

All authors listed have made a substantial, direct and intellectual contribution to the work, and approved it for publication.

## Conflict of Interest Statement

The authors declare that the research was conducted in the absence of any commercial or financial relationships that could be construed as a potential conflict of interest.
